# Cerebrospinal fluid leakage after radioisotope cisternography is not influenced by needle size at lumbar puncture in patients with intracranial hypotension

**DOI:** 10.1186/1743-8454-6-5

**Published:** 2009-05-27

**Authors:** Koichi Takahashi, Tatsuo Mima

**Affiliations:** 1 Sanno Hospital, Department of Neurosurgery, Akasaka 8-10-16, Minato-ku, Tokyo 107-0052, Japan

## Abstract

**Background:**

Radioisotope (RI) cisternography is considered to be the most important examination for the final diagnosis of intracranial hypotension, typically indicating cerebrospinal fluid (CSF) leakage as RI parathecal activity. Early bladder filling (EBF) of RI is another important finding. However, whether EBF without parathecal activity represents real CSF leakage due to intracranial hypotension or only an epiphenomenon of lumbar puncture causing CSF leak through a needle hole has been questioned.

**Methods:**

To address this issue, we performed quantitative analysis of RI cisternography on 171 patients with suspected intracranial hypotension using different needle sizes (22 G, 23 G and 25 G) and compared RI residual activity in the CSF at different time points after injection. We also analyzed occurrence of early bladder filling and post-lumbar puncture headache.

**Results:**

No significant difference in RI residual activity was identified between the 22 G, 23 G and 25 G groups. The incidence of parathecal activity and early bladder filling was not significantly different between groups. The 22 G and 23 G groups had a higher but non-significant incidence of post lumbar headache.

**Conclusion:**

The results suggest that needle size, at least for 22–25 G, does not affect the results of RI cisternographic diagnostic tests for CSF leakage and bladder filling in intracranial hypotension.

## Background

Intracranial hypotension has increasingly gained recognition as a pathophysiological entity since Mokri reported pachymeningeal gadolinium enhancement on magnetic resonance imaging (MRI) in low intracranial pressure headaches [1]. Intracranial hypotension is typically characterized by orthostatic headache and other clinical symptoms which have been identified by imaging techniques [2,3]. Some papers have reported recently that intracranial hypotension occurs after injury such as traffic accidents [4-7]. Although imaging features on MRI such as diffuse pachymeningeal enhancement and descent of the brain are important diagnostic findings [2,3,8-10] radioisotope (RI) cisternography is the gold standard for diagnosing intracranial hypotension in that it visualizes the circulation of cerebrospinal fluid (CSF) [2,3,8-14]. Typical imaging findings in cases of intracranial hypotension involve detection of parathecal activity (PTA) pointing to the level or approximate site of CSF leakage. The other common finding is the early appearance of radioactivity in the urinary bladder (early bladder filling; EBF) [2,3]. EBF is thought to indicate intrathecally introduced RI that has been extravasated and has entered the venous system with subsequent early renal clearance. However, some doubt remains as to whether findings of EBF are diagnostic of intracranial hypotension. It has been suggested that EBF without appearance of PTA could be due to lumbar puncture causing CSF leakage through a needle hole in the dura. To test this possibility, we performed quantitative analysis of RI cisternography using different needle sizes (22 G, 23 G and 25 G) and compared RI residual activity, or percentage activity remaining in the CSF spaces, between needle sizes. We also recorded early bladder filling and analyzed the occurrence of post-lumbar headache (PLH) in the same groups.

## Methods

### Patients

Intracranial hypotension was suspected in patients based on clinical signs, particularly orthostatic headache of unknown origin or subsequent to injury, and MRI and/or CT findings such as brain sagging in the convexity. Between June 2006 and September 2007, a total of 173 of those cases underwent RI cisternography by intrathecal lumbar injection of 1 ml (37 MBq at calibration time) of ^111^In (Nihon Medi-Physics, Tokyo, Japan) using a spinal needle (TERMO^R^; Tokyo, Japan). Two cases were excluded due to a misplaced injection into the epidural space. Hence 171 patients were subjected to the analysis in this study. Before conducting RI cisternography, written informed consent was obtained from all patients and ethical approval was obtained from our institution for the study.

### Procedures

Subjects were randomly divided into three groups based on the needle size used for lumbar puncture in radioisotope cisternography; 22 G (outer diameter (o.d.), 0.70 mm; inner diameter (i.d.), 0.41 mm), 23 G (o.d., 0.65 mm; i.d., 0.35 mm) or 25 G (o.d., 0.55 mm; i.d., 0.26 mm). Lumbar puncture was performed at lower lumbar level (mostly at L3/4 and occasionally at L2/3) in a lateral recumbent position under local anesthesia using 8 ml of 1% lidocaine.

Posteroanterior and anteroposterior whole-body planar scintigraphy was performed with a single-headed gamma camera (RC 2500 IV; Hitachi Medical, Tokyo, Japan) at 1, 3, 5 and 24 h after injection. The CSF space was scanned downward from the head at a speed of 8 cm/min. Patients were prohibited from urinating for 1 h after RI injection. Immediately after the first scan, patients were allowed to urinate, then again prohibited from urinating until after the next scan.

### Quantitative radioisotope cisterngraphy

**Figure 1:**
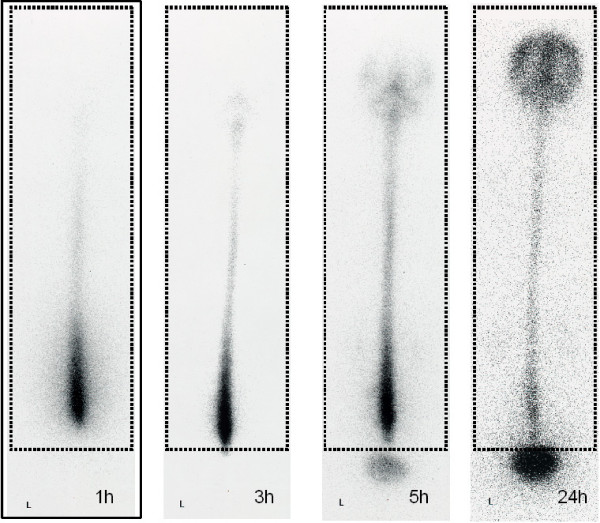
**Radioisotope cisternography images from a 36-year-old man at 1, 3, 5 and 24 h after RI injection**. Two regions of interest (ROI) were analyzed one covering the entire CSF space (dotted line) and the other the whole CSF space plus the urinary bladder (solid line). Quantitative analysis of RI cisternography was performed at 1, 3, 5 and 24 h after RI injection. RI residual activity at n hours after RI injection was calculated as follows: (RI activity in ROI of whole CSF space at n hours after injection/RI activity in ROI from head to urinary bladder at 1 h after injection) × 100. This patient showed late bladder filling at 5 h. L: left side.

Two regions of interest (ROI) were selected; one including the entire CSF space and the second including the CSF space plus the urinary bladder. (Figure 1). Quantitative analysis of RI cisternography was performed at 1, 3, 5 and 24 h after injection. Percentage RI residual activity in the CSF at n hours after RI injection was calculated as follows [9,13,14]:

(RI activity in ROI of the entire CSF space at n hours/RI activity in ROI of the whole region including the urinary bladder at 1 h) ×100

**Figure 2:**
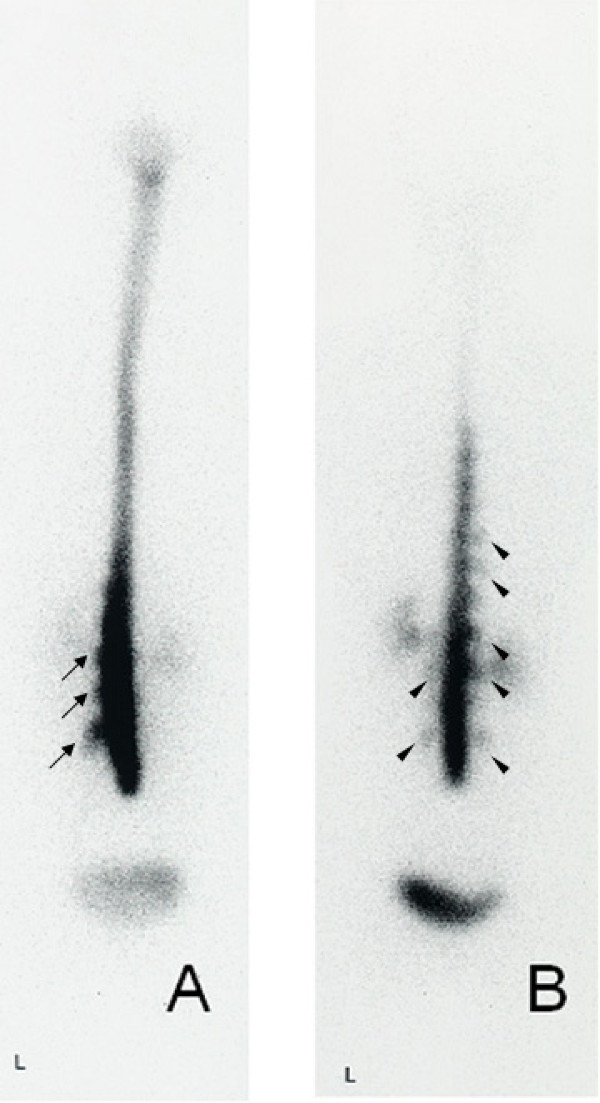
**Radioisotope cisternography images in two patients at 1 h after injection**. A) Parathecal activity at lumbar level (arrows). B) Parathecal activity at both lumbar and thoracic levels (arrowheads). L: left side.

**Figure 3:**
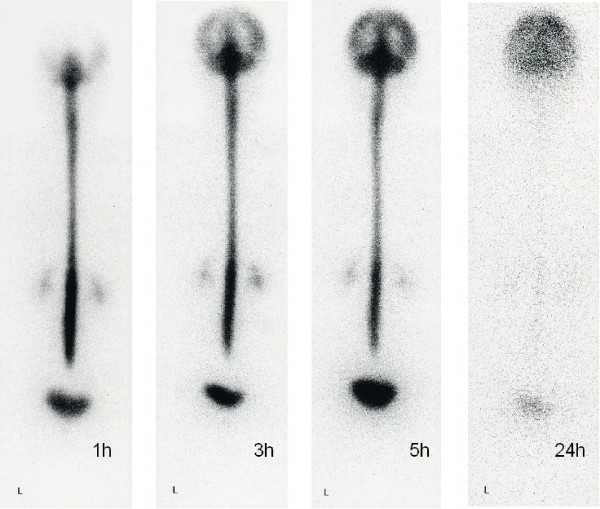
**The RI cisternography sequence from another patient showing early bladder filling at 1 h (EBF1)**. L: left side.

For assessment of RI excretion, RI activity was counted in each ROI for posteroanterior images. Findings from RI cisternography were classified into three groups: 1) clear detection of PTA showing direct proof of CSF leakage (PTA group); 2) EBF without detection of PTA or indirect proof of CSF leakage (EBF group), and 3) normal group. PTA was classified as occurring either at the lumbar level or at both lumbar and thoracic levels. (Figure 2). EBF was classified according to the duration from RI injection until detection of RI in the bladder, as EBF at 1 h (EBF1). (Figure 3) or EBF at 3 h (EBF3). Patients without PTA and with bladder filling at 5 h. (Figure 1) or no bladder filling even at 5 h were judged as having no CSF leaks and hence normal.

### Post lumbar-puncture headache

The severity of PLH was judge by questioning the patient 24 h after lumbar puncture. PLH was compared between groups, and the severity was graded in four categories as follows: PLH(2+), headache more severe than or equal to the most severe headache the patient had ever experienced; PLH(+), headache increased after lumbar puncture, but less severe than PLH(2+); PLH(-), headache of usual severity or less; or PLH(±), headache of usual severity or less headache, but with other aggravating symptoms such as more severe back pain, or lumbago.

### Data analysis

For statistical analysis of RI residual activity among 22 G, 23 G and 25 G groups, values were compared among the three groups using parametric, non-repeated measures by analysis of variance (ANOVA). For statistical analysis of frequency of PLH, values were compared between observed frequency and expected frequency using an m × n χ^2 ^test. Values of *P *< 0.05 were considered statistically significant. Excel^R ^statistical software (ystat 2006. xls; Igaku Tosho Shuppan, Tokyo, Japan) was used for all statistical analyses.

## Results

Two cases with misconducted RI cisternography were excluded and 171 cases were used for the analysis in the present study. The groups comprised 57 patients (26 male, 31 female) in the 22 G group, 57 patients (28 male, 29 female) in the 23 G group, and 57 patients (25 male, 32 female) in the 25 G group. Mean age was 37.7 years in the 22 G group, 37.6 years in the 23 G group and 38.1 years in the 25 G group, with no significant differences in gender or age between these groups. All had a history of pre-puncture headache.

### Radio isotope cisternography

**Figure 4:**
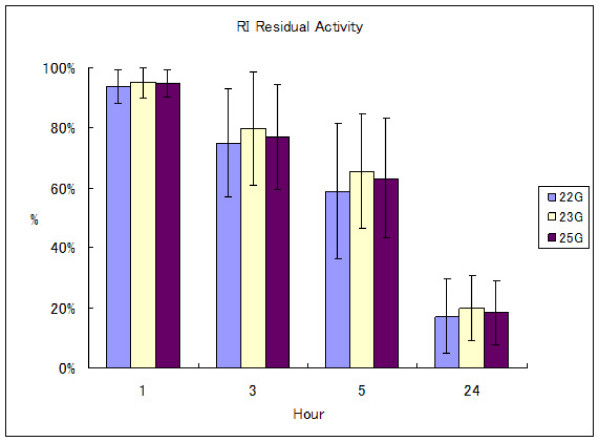
**Relationship between time after injection and calculated residual radioisotope activity in the CSF space among 22 G, 23 G and 25 G patient groups**. X axis shows time after RI injection. Y axis shows percentage RI residual activity. No significant differences between groups were identified at any time point.

Mean ± standard deviation (SD) RI residual activity for the 22 G group was 93.7 ± 5.6% at 1 h, 75.1 ± 18.0% at 3 h, 58.9 ± 22.6% at 5 h and 17.2 ± 12.5% at 24 h. For the 23 G group, RI residual activity was 95.0 ± 5.1% at 1 h, 79.8 ± 18.7% at 3 h, 65.5 ± 19.3% at 5 h and 19.9 ± 10.8% at 24 h. For the 25 G group, RI residual activity was 94.7 ± 4.6% at 1 h, 77.0 ± 17.4% at 3 h, 63.2 ± 20.1% at 5 h and 18.3 ± 10.7% at 24 h. (Figure 4). Although the 22 G group tended to show less RI residual activity, equating to more CSF leakage, compared to 23 G and 25 G group, no significant differences among these groups were identified by non-repeated measures ANOVA.

The pattern of RI cisternographic findings is shown in Table 1. RI cisternography revealed 16 PTA (28.1%; 11 lumbar level and 5 lumbar and thoracic levels) with 22 G, 10 PTA (17.5%; 9 lumbar level and 1 lumbar and thoracic levels) with 23 G, and 18 PTA (31.6%; 12 lumbar level and 6 lumbar and thoracic levels) with 25 G needles. All cases with PTA also had EBF at 1 h, indicating that the leaked CSF was immediately transferred into the blood circulation. Numbers of patients designated PTA, EBF1, EBF3, and normal were not significantly different between needle groups.

**Table 1:** Quantitative radioisotope cisternography in patients with intracranial hypotension.

	22 G (n = 57)		23 G (n = 57)		25 G (n = 57)	
PTA	16	28.1%	10	17.5%	18	31.6%
EBF 1	12	21.1%	6	10.5%	4	7.0%
EBF 3	16	28.1%	28	49.1%	26	45.6%
normal	13	22.8%	13	22.8%	9	15.8%

Data shows the number and percentage of patients in three groups after lumbar puncture with 22, 23, or 25 gauge needles. PTA: patients that showed radioisotope parathecal activity; EBF1: early bladder filling at 1 h without PTA; EBF3: early bladder filling at 3 h without PTA; normal: patients having cisternograms without PTA or EBF. All PTA patients had EBF at 1 h. There were no significant differences between needle sizes.

### Post-lumbar headache

Severe headache identified as PLH(2+) tended to occur more often with 22 G and 23 G than with 25 G needles (Table 2). However, no significant differences among groups were identified by m × n χ^2 ^test. Combined frequency of PLH(+) and PLH(2+) was 63.1% in the 22 G group, 64.9% in the 23 G group, and 49.2% in the 25 G group. Around half of the cases experienced PLH. The relation between the severity of PLH and residual activity in the CSF at different times after injection is shown in Table 3. Patients with more severe PLH tended to have less RI residual activity and hence more parathecal leakage and bladder filling, even though no significant differences were identified.

**Table 2:** Numbers and percentages of patients with intracranial hypotension that showed varying degrees of post-lumbar puncture headache after puncture with 22, 23, or 25 gauge needles

	22 G (n = 57)		23 G (n = 57)		25 G (n = 57)	
PLH (-)	15	26.3%	14	24.6%	20	35.1%
PLH (±)	6	10.5%	6	10.5%	9	15.8%
PLH (+)	26	45.6%	28	49.1%	27	47.4%
PLH (2+)	10	17.5%	9	15.8%	1	1.8%

PLH(-): headache of usual severity; PLH(±): headache plus other symptoms; PLH(+): headache increased; PLH(2+): headache very severe. Although severe headache occurred more frequently with the larger needles, the differences were not significant.

**Table 3:** The percentage residual radioisotope activity in the CSF (% ± SD) in patients with differing degrees of post lumbar headache at 1 h, 3 h, 5 h, and 24 h after injection.

	1 h	3 h	5 h	24 h
PLH (-) (n = 49)	95.0 (± 4.6)	83.0 (± 18.5)	71.3 (± 19.2)	22.0 (± 11.8)
PLH (±) (n = 21)	95.5 (± 5.9)	77.7 (± 10.6)	64.8 (± 13.1)	19.7 (± 9.7)
PLH (+) (n = 81)	94.3 (± 4.9)	74.3 (± 18.4)	59.0 (± 20.9)	17.0 (± 11.5)
PLH (2+) (n = 20)	93.2 (± 6.0)	74.8 (± 20.0)	54.7 (± 23.9)	14.7 (± 9.8)

PLH(-): headache of usual severity or less; PLH(±): headache of usual severity or less headache, but with other aggravating symptoms; PLH(+): headache increased after lumbar puncture; PLH(2+): headache more severe than or equal to the most severe headache ever experienced. The (+) and (2+) groups tended to have more CSF leakage but the differences were not significant.

## Discussion

Intracranial hypotension has gradually become recognized as a defined syndrome since Mokri reported characteristic features on MRI [1]. While CT myelography, MR myelography remain useful diagnostic modalities, RI cisternography is still the most important examination for the final diagnosis of intracranial hypotension. To the best of our knowledge, no reports have described RI cisternographic findings with quantitative analysis comparing differences in needle size. In this study, differences in needle size between the 22 G, 23 G, and 25 G groups did not significantly affect RI clearance from the CSF or the pattern of RI cisternographic findings, even though the 22 G group tended to show less RI residual activity. Subjects in this study were patients with suspected intracranial hypotension. Some patients had CSF leaks of differing degrees and others had no CSF leak. However, the number of cases in each needle size group was 57, which may be sufficiently large to eliminate CSF leakage discrepancies among groups. Our results may thus indicate that volume of CSF leakage through needle holes is much smaller than real CSF leakage causing intracranial hypotension.

According to previous reports regarding occurrence of PLH, a larger needle gives rise to PLH more frequently [15-18]. Our data also showed PLH was more frequent in the 22 G and 23 G groups than in the 25 G group. In addition, the 22 G group tended to show more rapid RI clearance, but these differences were not significant. Increased CSF leak caused by lumbar puncture might plausibly result in greater incidence of PLH, even though the volume of leakage is small. Our results suggest that CSF leakage through a needle hole from lumbar puncture can thus be considered negligible for diagnosis of intracranial hypotension.

PLH is a common complication following lumbar subarachnoid anesthesia, with frequencies of 12–38% reported [15-19]. This headache is decidedly postural, beginning when the patient sits or stands upright and relieved when the patient is recumbent. Severe headaches are often associated with nausea, blurred vision, vertigo and back pain. Kuntz *et al*. reported that existing headache preceding lumbar puncture, a younger age, and female gender represented high-risk factors for developing PLH [19]. PLH occurred more frequently in this study than in previous studies [15-19]. This was probably due to differences in study objectives. Previous reports have mainly been based on cases of spinal anesthesia that were considered to have no CSF leakage, while our cases all involved patients with suspected intracranial hypotension. This difference in PLH incidence suggests that patients with intracranial hypotension may be more sensitive to additional CSF leak caused by lumbar puncture.

## Conclusion

The needle size from 22 G to 25 G used for lumbar injection did not affect the rate of CSF leakage or distribution on RI cisternography in patients with symptoms of intracranial hypotension. Although post-lumbar headache tended to occur more often in the 22 G and 23 G groups compared to the 25 G group, no significant differences were identified. Our results also suggest that the risk of CSF leakage through a needle hole from lumbar puncture can be considered negligible when used for diagnosis of intracranial hypotension.

## Abbreviations

EBF: early bladder filling; MRI: magnetic resonance imaging; PLH: post-lumbar headache; PTA: parathecal radioactivity; RI: radioisotope cisternography.

## Competing interests

The authors declare that they have no competing interests.

## Authors' contributions

KT and TM contributed equally to this work. KT performed data acquisition and analysis and drafted the manuscript. TM designed this clinical study. KT and TM have read and approved the final version of the manuscript.

## References

[B1] Mokri B, Parisi JE, Scheithauer BW, Piepgras DG, Miller GM (1995). Meningeal biopsy in intracranial hypotension: meningeal enhancement on MRI. Neurology.

[B2] Mokri B (2004). Low cerebrospinal fluid pressure syndromes. Neurol Clin N Am.

[B3] Schievink WI (2006). Spontaneous spinal cerebrospinal fluid leaks and intracranial hypotension. JAMA.

[B4] Ishikawa S, Yokoyama M, Mizobuchi S, Hashimoto H, Moriyama E, Morita K (2007). Epidural blood patch therapy for chronic whiplash-associated disorder. Anesth Analg.

[B5] Shinonaga M, Suzuki S (2003). Diagnosis and treatment of traumatic intracranial hypotension (cerebrospinal fluid hypovolemia). Neurotraumatology.

[B6] Shinonaga M (2005). Chronic headache attributed to CSF hypovolemia (broadening of CSF hypotension. Cephalalgia.

[B7] Shinonaga M (2007). Cerebrospinal fluid hypovolemia as a cause of post-traumatic syndrome. Cephalalgia.

[B8] Grimaldi D, Mea E, Chiapparini L, Ciceri E, Nappini S, Savoiardo M, Castelli M, Cortelli P, Carriero MR, Leone M, Bussone G (2004). Spontaneous low cerebrospinal pressure: a mini review. Neurol Sci.

[B9] Horikoshi T, Ikegawa H, Uchida M, Takahashi T, Watanabe A, Umeda T (2006). Tracer clearance in radionuclide cisternography in patients with spontaneous intracranial hypotension. Cephalalgia.

[B10] Miyazawa K (2004). Diagnosis and therapy of spontaneous intracranial hypotension syndrome. No To Shinkei.

[B11] Ali SA, Cesani F, Zuckermann JA, Nusynowitz ML, Chaljub G (1998). Spinal-cerebrospinal fluid leak demonstrated by radiopharmaceutical cisternography. Clin Nucl Med.

[B12] Chung SJ, Kim JS, Lee MC (2000). Syndrome of cerebral spinal fluid hypovolemia: clinical and imaging features and outcome. Neurology.

[B13] Horikoshi T, Uchida M, Watanabe A, Ikegawa H, Umeda T (2006). Jugular compression and radionuclide cisternographic patterns in patients with chronic headache. Headache.

[B14] Moriyama E, Ogawa T, Nishida A, Ishikawa S, Beck H (2004). Quantitative analysis of radioisotope cisternography in the diagnosis of intracranial hypotension. J Neurosurg.

[B15] Raskin (1990). Lumbar puncture headache: a review. Headache.

[B16] Rasmussen BS, Blom L, Hansen P, Mikkelsen SS (1989). Postspinal headache in young and elderly patients. Two randomised, double-blind studies that compare 20- and 25-gauge needles. Anaesthesia.

[B17] Tourtellotte WW, Haerer AF, Hellar GL, Somers JE (1964). Post lumbar puncture headaches.

[B18] Vandam LD, Dripps RD (1960). Long-term follow-up of patients who received 10,098 spinal anesthetics. IV. Neurological disease incident to traumatic lumbar puncture during spinal anesthesia. J Am Med Assoc.

[B19] Kuntz KM, Kokmen E, Stevens JC, Miller P, Offord KP, Ho MM (1992). Post-lumbar puncture headaches: experience in 501 consecutive procedures. Neurology.

